# Bilateral Knee Pain Associated with Bone Infarction in a Patient with Behcet's Disease

**DOI:** 10.1155/2012/539310

**Published:** 2012-11-08

**Authors:** Pelin Oktayoglu, Figen Cevik, Mehmet Tahtasiz, Serda Em, Mehtap Bozkurt, Ahmet Kapukaya, Kemal Nas

**Affiliations:** ^1^Department of Physical Medicine and Rehabilitation, Faculty of Medicine, Dicle University, 21280 Diyarbakir, Turkey; ^2^Department of Physical Medicine and Rehabilitation, Diyarbakir State Hospital, 21280 Diyarbakir, Turkey; ^3^Department of Orthopaedics and Traumatology, Faculty of Medicine, Dicle University, 21280 Diyarbakir, Turkey

## Abstract

We describe a 31-years-old female patient with severe pain in both knees who had been diagnosed as Behcet's disease (BD) for 12 years. She had had a history of complications due to BD including superior vena cava thrombosis, pulmonary thromboembolism, uveitis, and erythema nodosum and has reported the administration of corticosteroid therapy irregularly. After radiologic evaluation, she has been diagnosed with bone infarction of both left and right knee with the existance of lupus anticoagulants (LA) positivity. Severe joint pain without the evidence of arthritis must alert the clinician to the possibility of bone necrosis of the extremity, although those may rarely occur bilateral in BD.

## 1. Introduction

Osteonecrosis is a disease in which death of cellular elements of bone occurs as a result of diminished arterial blood supply. Immunologic factors, vasculitis, disease-associated features, as well as antiphospholipid antibodies, and prolonged use of corticosteroids have been involved in the development of osteonecrosis [1]. Bone infarction is a term referring to osteonecrosis that exists metaphyseal or diaphyseal region of long bones [2, 3]. Coagulation abnormalities and intravascular thrombosis might represent risk factors for the development of bone necrosis by predisposing patients to thromboembolic events [4]. Bone infarctions and osteonecrosis are rare causes of musculoskeletal pain in patients with BD [3] but corticosteroid therapy and presence of lupus anticoagulants may trigger potential mechanisms those result with bone infarctions or osteonecrosis in patients with BD.

## 2. Case Report 

**Figure 1:**
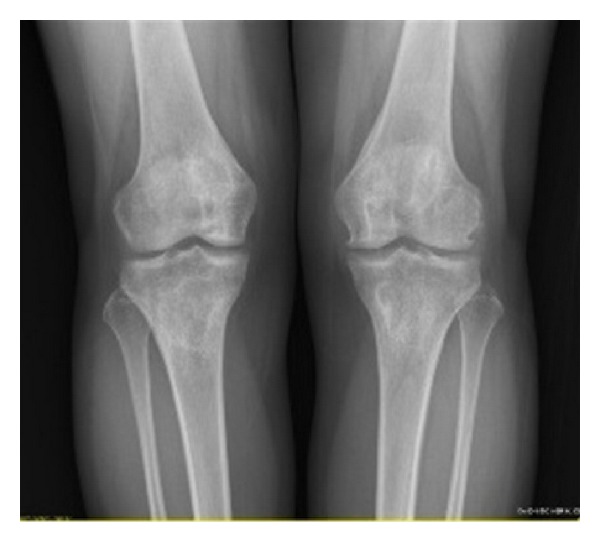
Density changes, sclerosis with cyst like formations in bony trabecula at distal femur and proximal tibia in AP radiogram.

A 31-year-old female patient has presented with insidious pain in her two knees for two years that has exacerbated within the past two months. In her history, she was diagnosed as Behcet's disease 12 years ago. Initially, oral ulcerations, genital ulcerations, and Pathergy test were positive. She was medicated with colchicine 1.5 mg/day by the dermatologists at the initiation of her illness. Two years later, she had a posterior uveitis attack. She also had a history of pulmoner thromboembolism and thrombosis in superior vena cava. At this time she was treated with monthly cyclophosphamide administration by rheumatologists, but she discontinued the therapy with individual reasons and medicated with azathioprine and colchicine with concomitant prednisolone therapy. She also had a history of erythema nodosum like lesions. Arthritis was also described in her left knee for several times and treated with nonsteroidal anti-inflammatory drugs. There was no history of miscarriage, use of oral contraceptives but there was a history of smoking five cigarettes a day. By physical examination, we have revealed no aphthous or genital ulceration and have found no limitation of motions and no signs of synovitis in joints, but only tenderness over the two knees were recognized. Skin lesions were not observed. Laboratory tests were as follows, WBC 5250, hematocrit 36.3%, platelet 383000/mm^3^, total protein 8.3 g/dL, albumin 4 g/dL, AST 15 IU/L, ALT 14 IU/L, creatinine 0.58 mg/dL, alkaline phosphatase 48 IU/L, and parathyroid hormone 36.82 pg/mL. Erythrocyte sedimentation rate was 46 mm/hour, and C-reactive protein was 0.58 mg/dL. Thyroid hormone levels, urinalysis, and coagulation tests were negative. Protein C and Protein S values were normal. Anticardiolipin IgG and IgM, rheumatoid factor, antinuclear antibody, and anti-dsDNA were also negative. Lac Confirm was 56.1 sec (30–38 normal ranges) Lac Screen was 51.9 (31–44 normal ranges). In conventional radiography, there were density changes, sclerosis with cyst like formations in bony trabecula at distal femur and proximal tibia of two extremities (Figure 1). 

**Figure 2:**
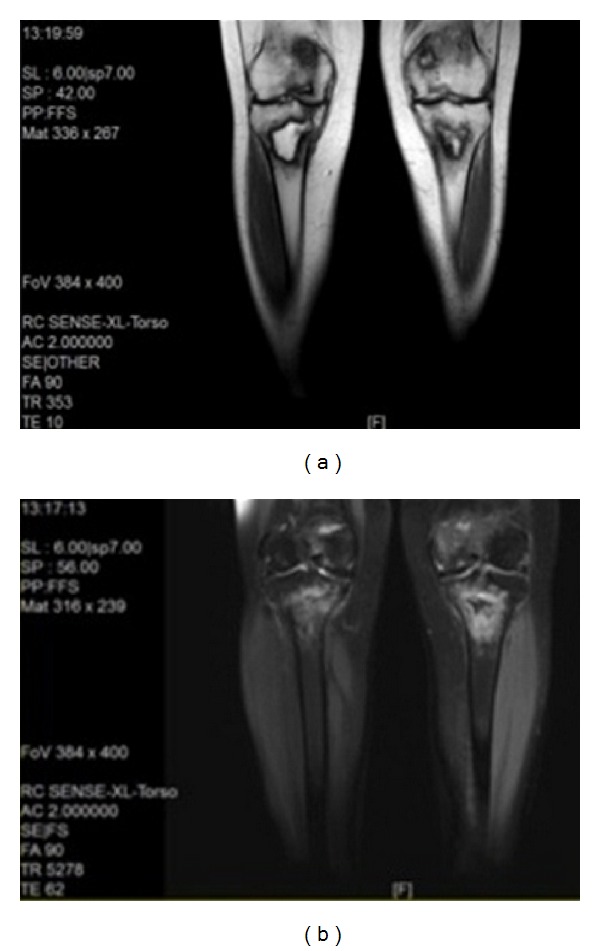
Signal changes in distal femur and metaphyseal-diaphyseal region of tibia consists with bone infarction in T1 (a) and T2 (b) weighted images.

Magnetic resonance imaging revealed bilateral signal changes in distal femurs and metaphyseal-diaphyseal region of tibias that were consistent with bone infarctions in these areas (Figures 2(a) and 2(b)).

She was diagnosed as BD with severe bone infarction. She was recommended to continue a period of rest and indomethacin therapy. After two weeks, joint pain has reduced. 

## 3. Discussion

BD is characterized by several systemic manifestations as well as vascular involvement. Most clinical manifestations are related to underlying vasculitides. BD is differentiated from other vasculitis with involving blood vessels of all sizes and involving both arterial and venous sides of circulation. In most cases, vasculitis is connected in the pathogenetic mechanism of bone necrosis [1]. On the other hand, antiphospholipid antibodies are associated with vessel thromboses of all sizes [5, 6] thus, a thrombotic microvasculopathy at the terminal arteries of bone supports the critical role of these antibodies in the pathogenesis of osteonecrosis [5]. LA is a member of antiphospholipid antibodies and plays an unclear role in coagulation system. Nagasawa et al. retrospectively investigated 111 patients with SLE for osteonecrosis [7]. The percentage of patients who had LA was greater among those with osteonecrosis than among those without. In another study Vayá et al. investigated thrombophilic risk factors in patients with SLE and showed that the patients with thrombosis showed significantly higher percentage of lupus anticoagulants compared to patients without thrombosis [8]. Bone infarction involving two extremities in BD is very rare in literature [3, 9]. The unknown role of LA in thrombotic events and history of corticosteroid medication may trigger bone infarctions; however, we have found no reports about the relationship between LA and bone infarction in patients with BD. Severe joint pain without the evidence of arthritis must alert the clinician related with the risk of bone infarctions; however, it rarely occurs bilateral in patients with BD as observed in our patient.
